# Benchmarking ensemble docking methods in D3R Grand Challenge 4

**DOI:** 10.1007/s10822-021-00433-2

**Published:** 2022-02-24

**Authors:** Jessie Low Gan, Dhruv Kumar, Cynthia Chen, Bryn C. Taylor, Benjamin R. Jagger, Rommie E. Amaro, Christopher T. Lee

**Affiliations:** 1 San Diego Jewish Academy, San Diego, 92130 CA USA; 2 Present Address: California Institute of Technology, Pasadena, CA 91125 USA; 3 Rancho Bernardo High School, San Diego, CA 92128 USA; 4 Present Address: University of California Berkeley, Berkeley, CA USA; 5 Canyon Crest Academy, San Diego, CA 92130 USA; 6 Department of Chemistry and Biochemistry, University of California San Diego, La Jolla, CA 92093 USA; 7 Present Address: Discovery Sciences, Janssen Research and Development, San Diego, CA 92121 USA; 8 Present Address: Department of Bioengineering and Therapeutic Sciences, University of California San Francisco, San Francisco, CA 94158 USA; 9 Department of Mechanical and Aerospace Engineering, University of California San Diego, La Jolla, CA 92093 USA

**Keywords:** Computational biophysics, Ensemble docking, Molecular dynamics, Drug discovery, Restrained docking

## Abstract

The discovery of new drugs is a time consuming and expensive process. Methods such as virtual screening, which can filter out ineffective compounds from drug libraries prior to expensive experimental study, have become popular research topics. As the computational drug discovery community has grown, in order to benchmark the various advances in methodology, organizations such as the Drug Design Data Resource have begun hosting blinded grand challenges seeking to identify the best methods for ligand pose-prediction, ligand affinity ranking, and free energy calculations. Such open challenges offer a unique opportunity for researchers to partner with junior students (e.g., high school and undergraduate) to validate basic yet fundamental hypotheses considered to be uninteresting to domain experts. Here, we, a group of high school-aged students and their mentors, present the results of our participation in Grand Challenge 4 where we predicted ligand affinity rankings for the Cathepsin S protease, an important protein target for autoimmune diseases. To investigate the effect of incorporating receptor dynamics on ligand affinity rankings, we employed the Relaxed Complex Scheme, a molecular docking method paired with molecular dynamics-generated receptor conformations. We found that Cathepsin S is a difficult target for molecular docking and we explore some advanced methods such as distance-restrained docking to try to improve the correlation with experiments. This project has exemplified the capabilities of high school students when supported with a rigorous curriculum, and demonstrates the value of community-driven competitions for beginners in computational drug discovery.

**Supplementary Information:**

The online version contains supplementary material available at 10.1007/s10822-021-00433-2.

## Introduction

Drug discovery efforts often require the screening of many compounds to determine their efficacy. Owing to the high cost of experimental screening and advances in computer models, the use of inexpensive computational screening methods to enrich compounds in large datasets have been used in drug discovery pipelines for several decades [1]. Historically, initial compound libraries contained only a few ‘actives’ among many orders of magnitude more inactive compounds. Computer-aided drug discovery (CADD) methods, such as virtual screening, could be used to filter out unlikely candidates, reducing experimental costs, and accelerating the initial discovery phase [2–4]. The drug discovery paradigm fundamentally shifted in 2018 when Enamine Ltd. [5] partnered with ZINC [6] to provide the community with access to their REAL database of more than 300 million synthetically feasible chemical structures (today, the Enamine REAL library contains nearly 2 billion structures). This database and others like it have increased available chemical matter by more than an order of magnitude but produced another challenge: how to sift through the hundreds of millions (now billions) of virtual compounds. Instead of desperately looking for a single active compound and being concerned with missing false negatives, the primary objective of many CADD methods is to reduce the rate of false positives and minimize the number of hits to test experimentally, reducing experimental costs and increasing the likelihood of finding a drug candidate [7, 8].

Due to the diversity and breadth of the CADD research community, many methods have been developed. Cross-comparison and benchmarking between the different approaches is necessary for identifying the limitations of the docking method and areas for improvement. The Drug Design Data Resource (D3R) hosts blinded community prediction challenges to evaluate these software and techniques and compare their effectiveness on benchmark systems, such as the HSP90 chaperone protein, the farnesoid X receptor, and the Cathepsin S protease (CatS) [9–11]. In 2018, D3R hosted Grand Challenge 4 (GC4), which had components of pose prediction, free energy prediction, and ligand affinity rank ordering [12].

We participated in Subchallenge 2, a ligand affinity ranking challenge for the Cathepsin S protease with a set of 459 ligands provided by Janssen Pharmaceuticals [12]. CatS is a cysteine protease involved in the presentation of antigens by the MHC class II molecules within CD4^+^ T cells [13]. This makes it a promising target in autoimmune disease and allergy treatment, where inhibition of the immune response is critical for effective therapy [14–16].

We used molecular docking, a popular method of virtual screening, in a strategy known as the Relaxed Complex Scheme to account for protein flexibility [17]. Molecular docking applies a conformational search algorithm paired with an inexpensive, and often empirical, scoring function to find favorable lead compounds [18, 19]. By forgoing rigorous dynamics and detailed potential energy functions, such as those used in free energy calculations, docking approaches are designed to yield results quickly albeit with lower accuracy [20]. The speed of molecular docking codes enables the screening of hundreds of thousands to millions of compounds [21]. A risk of docking is the increased likelihood of false negatives. To this end, much work has been done by the community to develop improved algorithms which improve docking accuracy with minimal impact on speed [3].

In docking studies, proteins are represented as static structures [20, 22, 23]. To incorporate ligand flexibility, multiple ligand positions can be sampled through rotational torsions, i.e. conformer generation [24]. However, molecular dynamics (MD) simulations have revealed that thermal protein fluctuations in solute-based environments can give rise to varying conformational states, resulting in different binding sites [25]. Accounting for receptor binding site flexibility in molecular docking is a significant challenge. One solution is to perform ensemble docking. This involves docking a ligand compound library to a number of distinct, rigid receptor conformations to identify the receptor conformation that is best suited for that particular ligand (i.e. best docking score) [24, 26–28].

Here, we perform MD simulations of the receptor protein, CatS, to obtain unique conformational states and introduce structural variation in the binding site. MD simulations allow the exploration of multiple conformations of the protein while in a solute-based, native environment [29, 30]. This concept of selecting naturally-occurring conformations through MD for ensemble docking is known as the Relaxed Complex Scheme [30–35]. MD-generated ensembles of flexible binding sites have been used successfully in a number of studies to identify lead compounds [17, 36–39].

Incorporating more receptor conformations can increase the number of false positives and also increases the computational cost, as a complete docking protocol must be performed for each conformation. To address this, the trajectory can be clustered to extract unique, representative conformations [40, 41]. This methodology is still susceptible to the conformational sampling problem of MD, due to the large discrepancy between the accessible timescales of MD simulation (microseconds) and the slow, native dynamics of proteins (milliseconds and longer) [42, 43]. Although a trajectory may not statistically converge to encompass all possible conformations, studies have shown that clustering MD trajectories can reveal previously unknown druggable pockets [36].

Many studies have successfully used clustering methods in ensemble docking to extract relevant conformations, such as those based on root-mean-square deviation (RMSD) [30, 38], QR factorization [17, 37], and active pocket volume [30]. However, choosing the most appropriate clustering method for a system is still challenging and often dependent on human intuition.

Although ensemble docking has been successfully used to identify lead compounds, clustering methods in ensemble docking have not been extensively studied [44]. We explored three clustering methodologies in this study to investigate if they could (i) provide an accurate ligand ranking and (ii) give insights into CatS ligand binding mechanisms. The three clustering methods we used are: (1) Time-lagged Independent Components Analysis and K-means clustering (TICA) [45, 46], (2) Principal Component Analysis and K-means clustering (PCA) [45, 46], and (3) GROMOS RMSD clustering (GROMOS) [47]. TICA identifies the slowest motions of the simulation and projects the input features into a slow subspace where distinct clusters are kinetically separated [48]. PCA, on the other hand, finds features with the largest variance [49]. Lastly, GROMOS is a RMSD-based clustering method that counts the neighbors in a cluster based on a pre-set cutoff value and defines trajectory clusters by structural variation [47].

In this work, we apply the Relaxed Complex Scheme with these clustering methods and compare the ensemble docking results [34]. We test the accuracy of a state-of-the-art docking software, Schrödinger Glide [50]. We found that CatS is a difficult target for molecular docking and we explore some advanced methods such as distance-restrained docking to try to improve the correlation with experiments.

This manuscript presents the work of high school students who performed this work after completing BioChemCoRe, a 7 week crash course on computational chemistry (http://biochemcore.ucsd.edu/). These results help to illustrate the benefits and possibilities of teaching science as we do science [51, 52]. By participating in structured challenges with real-world significance, students gain motivation, confidence, and both technical and soft skills. Moreover, the exposure to the rigors of the scientific approach and the methods employed in the field of study aids them with their future career decisions. On the other hand, community-driven competitions and resources such as D3R’s Grand Challenge 4 can also benefit from student participation. Rarely do these programs receive submission which test the basic hypothesis. For example, is domain expertise required for the application of the methods of interest? Given the current state of tutorials or instructions available to the public, can students with limited domain experience use these resources to produce results without major technical difficulties? We posit that student participation can not only yield important benchmarking data but also serve to improve the documentation of our tools and methods.

## Materials and methods

**Fig. 1:**
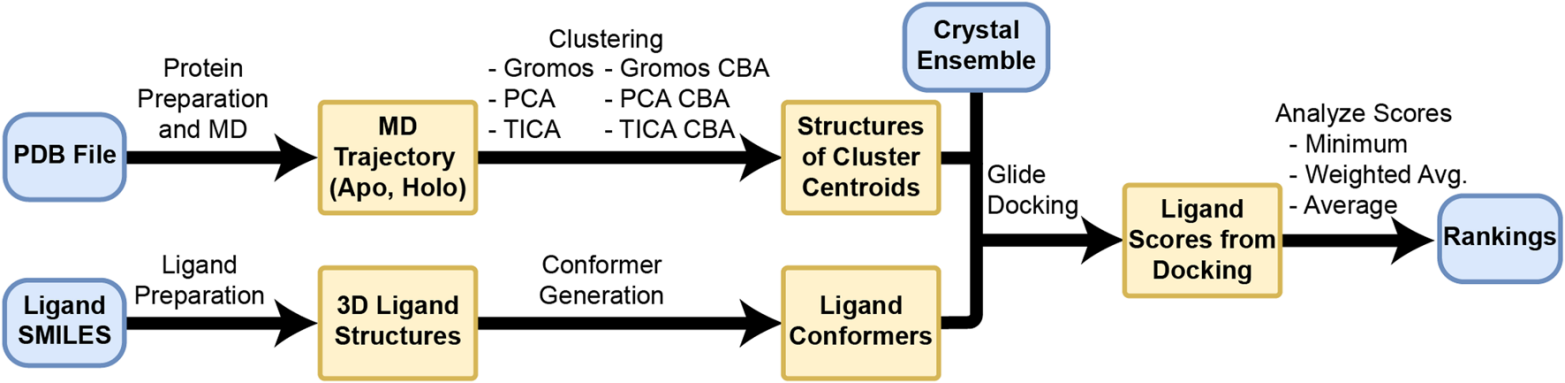
Workflow of the ensemble docking approach. A PDB file was selected and simulated using molecular dynamics. The resultant trajectory was clustered using six different methods, and cluster centroids were extracted as representative structures. Ligand SMILES were prepared as 3D structures and conformers were generated. Molecular docking of ligands was performed with Glide to the cluster centroids and the crystal ensemble. Pose scores were used to generate rank orderings and Kendall’s *τ* values when compared to the experimental rank ordering

All scripts used in this work can be found online at https://github.com/ctlee/bccgc4. A workflow of the methods is shown in Fig. 1.

### Molecular dynamics

A crystal structure of CatS (PDB ID: 5QC4 [15]) was obtained from the RCSB PDB database [53]. The structure was chosen due to its resolution of 2 Å and similarity of the cocrystallized ligand to those in the D3R dataset. This cocrystal was part of D3R’s prior Grand Challenge 3 (GC3), subchallenge 1, and the ligands in the CatS subchallenge of Grand Challenge 4 all contain the tetrahydropyrido-pyrazole core that 22 of the 24 ligands had in the previous challenge [11].

Models with (holo) and without (apo) the cocrystallized ligand were prepared for MD simulations. For both apo and holo models the same steps were performed with a few deviations noted below. Chain A of the structure was prepared in Schrödinger Maestro 2019 [54] with the Protein Preparation Wizard. For the holo simulation, the cocrystallized ligand was retained. For the apo simulation the ligand was removed [55]. Force field parameters for the ligand were derived from GAFF [56] with partial charges fit using the restrained electrostatic potential method (RESP) [57] from potentials computed using the AM1-BCC semi-empirical quantum mechanical method [58, 59]. For both systems, the protein termini were capped with an acetyl (ACE) and N-methyl amide (NME) capping groups. PROPKA [60, 61] was used to assign residue protonation states in a solvent of pH 5.0, to mimic experimental conditions of CatS binding assays [13]. Crystal waters with more than 2 hydrogen bonds to non-waters were retained.

Using a combination of pdb4amber and tleap from the AMBER 18 software suite, we parameterized the systems with the AMBER FF14SB forcefield, and solvated the systems with TIP4P-Ew up to a 15 Å buffer distance [62]. We added ions according to the SLTCAP tool by Schmit et al. [63] at 100 mM salt concentration, again to mimic experimental conditions [13].

All-atom, explicit-solvent MD simulation was performed using AMBER18 in four stages: minimization, heating, equilibration, and production [62]. The systems were gradually minimized in four steps: (i) minimization of only protons, restraining the protein and solvent, (ii) minimization of the solvent, restraining the protein, (iii) minimization of the protein sidechains, restraining the protein backbone, and (iv) minimization of all atoms. Restrained heating was performed in two steps: first, in the NVT ensemble the temperature was increased from 0 to 100 K over 50 ps using a Langevin thermostat, and second in the NPT ensemble the temperature was increased from 100 to 300 K over 200 ps using a Langevin thermostat while pressure was maintained at 1 bar using a Berendsen barostat. Equilibration was also performed in two stages, first with a restrained backbone, and second without restraints. For both equilibration stages the temperature was maintained at 300 K using a Langevin thermostat. For the restrained equilibration stage, 500 ps were run with a Berendsen barostat to equilibrate pressure to 1 bar. In the unrestrained equilibration step 1000 ps were run using a Monte Carlo barostat at 1 bar.

Production simulations were run in the NPT ensemble with the same conditions as the unrestrained equilibration step. Five independent simulations of each condition, apo and holo, of length 2 μs were run, totaling 20 μs (Fig.  S5). 450,000 frames total were saved per simulation type (450,000 for apo and 450,000 for holo). Hydrogen Mass Repartitioning (HMR) was performed with PARMED [62, 64] for all systems permitting a 4 fs timestep. Simulations were run with SHAKE restraints [65] and a non-bonded cutoff of 10 Å. All MD simulations were run on the Comet supercomputer at SDSC.

### Clustering

**Fig. 2:**
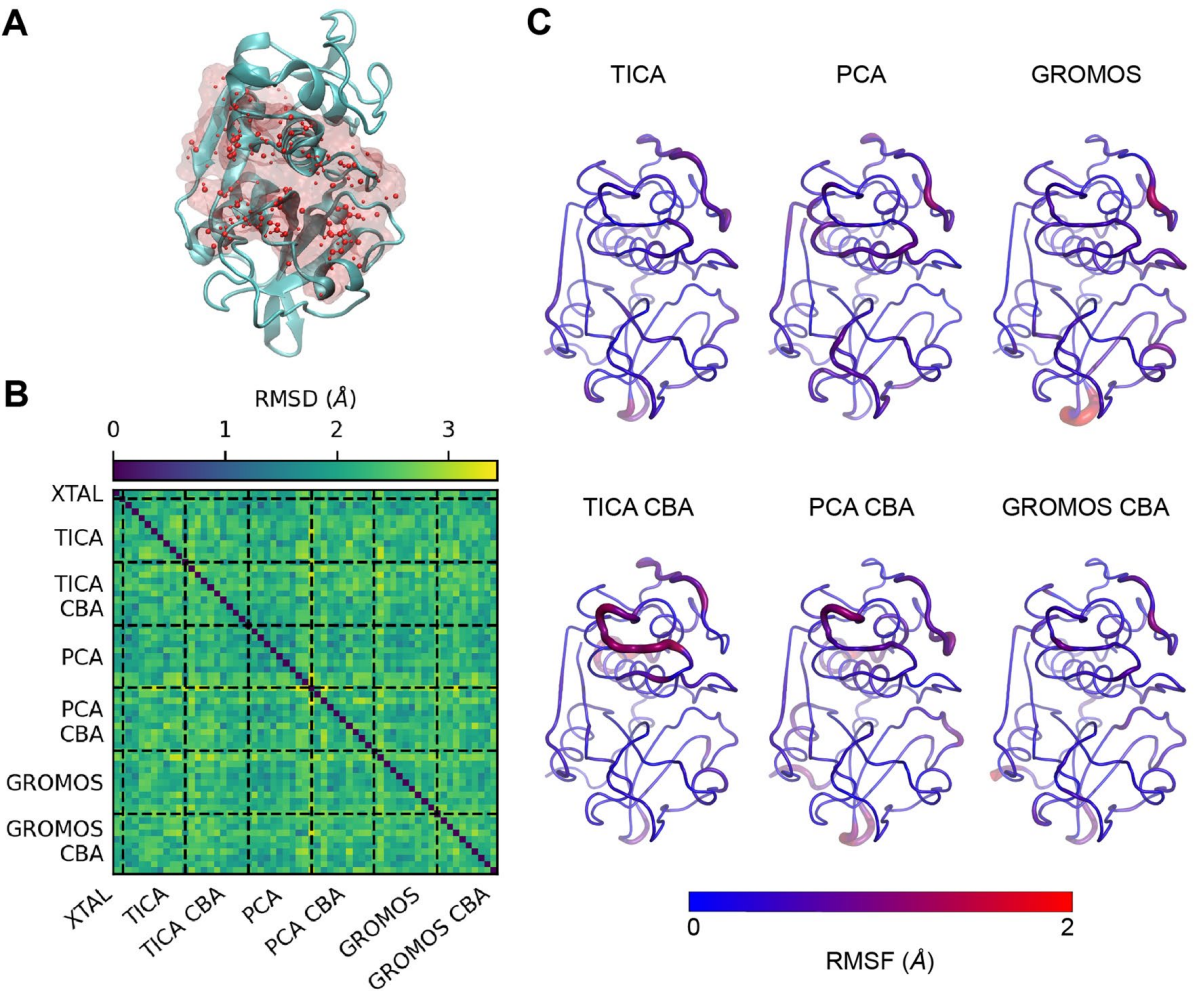
Apo molecular dynamics (MD) clustering results. **A** Binding Atoms definition for Clustered by Binding Atoms (CBA) centroids, defined by taking all atoms within 2 Å of docked poses of a ligand from the D3R dataset (‘CatS_2’, the second ligand in the dataset) from Glide apo blind docking. The crystal structure protein is depicted in NewCartoon and colored teal, while the binding atoms are both represented by red spheres and a transparent red surface representation, visualized in Visual Molecular Dynamics (VMD) [80, 81]. **B** The pairwise root-mean-square deviations (RMSDs) of the binding atoms of the crystal structure and all 10 centroid structures from each clustering method are depicted in a heatmap. The centroids obtained from clustering have a range of RMSDs and therefore have structural variability. **C** MD clustering extracts various centroid structures, and different clustering methods yield different conformations. The RMSF of the 10 centroids extracted from each clustering method, shown as the relative thickness and color, was calculated with MDTraj [71] and visualized using PyMOL [73]. The orientation of the protein for parts A and C are the same

The MD trajectory was clustered using three different clustering methods: (1) TICA and k-means [45, 46, 66, 67] on the protein backbone atom position coordinates, (2) PCA and k-means on the protein backbone atom position coordinates, and (3) GROMOS [47] on the C-alpha atom position coordinates. To identify a good set of initial input features, we compared the mean 10-fold cross-validated Variational Approach for Markov Processes (VAMP2) scores for three selections: (i) protein backbone atom positions, (ii) protein backbone torsions, and (iii) the positions of a binding atoms selection [68]. We decided to use the positions of protein backbone atoms because it had the largest VAMP2 score, indicating greater kinetic variance. The binding atoms were defined by taking all receptor atoms within 2 Å of the initial docked poses of a ligand from the D3R data-set. PCA was clustered on the same subset of backbone atom positions [45], and GROMOS was clustered on the C-alpha positions due to memory limitations. After the challenge, the clustering was reevaluated and a second discretization using the binding atoms selection, referred to as Clustered by Binding Atoms (CBA), was generated. We used similar ideas as the approach taken in Ref. [69], focusing on the binding site’s structural fluctuations rather than the entire structure. All six clustering methods (TICA, PCA, and GROMOS for backbone/C-alpha atoms and CBA) were also performed on the holo MD trajectories. The cluster centroids from the apo MD trajectory were compared by pairwise RMSD, using MDTraj and NumPy to calculate RMSDs and visualized using matplotlib [70–72] (Fig. 2B). They were also compared in terms of root-mean-square fluctuation (RMSF) to investigate the particular structural variability, computed in MDTraj and visualized in PyMOL [71, 73] (Fig. 2C). Trajectory frame indices for each cluster centroid are presented in Table S1. Frames were not minimized upon extraction.

#### Time-lagged independent components analysis and K-means (TICA)

TICA clustering was employed to capture the slow motions within the trajectory. TICA was performed with a lag time of 4 ps and a variance cutoff of 0.95 on the protein backbone atom coordinates [45]. The trajectory was projected into the TIC basis and the k-means algorithm was subsequently used to cluster the trajectory into 10 distinct clusters. The number of clusters was chosen upon inspecting the projected data. The 10 configurations from the trajectory, in real space, closest in TIC space to the cluster centroids were used for docking [46].

#### Principal components analysis and K-means (PCA)

PCA with a variance cutoff of 0.95 was performed on the protein backbone atom coordinates to capture large motions within the trajectory [45]. The trajectory was projected into the PC basis and the k-means algorithm was subsequently used to cluster the trajectory into 10 clusters. The number of clusters was chosen upon inspecting the projected data. The 10 configurations from the trajectory, in real space, closest in PC space to the cluster centroids were used for docking [46].

#### GROMACS RMSD-based clustering (GROMOS)

GROMOS clustering was performed on the alpha carbons in the protein to identify structurally diverse conformations according to RMSD [47]. The trajectories input to GROMOS were subsampled to yield frames every 0.4 ps. This was due to computational intractability at more frequent frame rates. The clustering RMSD cutoff was chosen to satisfy the following criteria: (i) the first cluster had less than 70% of the frames, (ii) the first 10 clusters contained at least 80% of the frames, and (iii) each of the first 10 clusters had at least 20 frames. A cutoff of 0.08 Å was used when clustering with alpha carbons while a cutoff of 0.15 Å was used when compared to CBA for the apo trajectory. A cutoff of 0.07 Å was used when clustering with alpha carbons while a cutoff of 0.135 Å was used when compared to CBA for the holo trajectory.

### Crystal ensemble

To compare the docking results between our ensemble of molecular dynamics derived structures with an ensemble of crystal structures, we prepared an ensemble of publicly available CatS cocrystal structures. The 55 structures were obtained by filtering Uniprot for Molecule Name “Cathepsin S” and the species Homo Sapiens. The 55 structures were prepared using Schrödinger Maestro 2019 in the same manner as we prepared PDB ID 5QC4 in “Molecular dynamics” section. Although we retained the ligands during preparation of the crystal ensemble, the ligands were ignored by selecting only the receptor when docking in Glide.

### Docking

Schrödinger Glide was used to dock the 459 ligands in the D3R GC4 CatS challenge to the centroids of the clustered MD trajectory, the original crystal structure PDB ID 5QC4, and the ensemble of 55 cocrystal structures [50, 74]. In addition, many iterations of Glide docking were run with modifications to further improve the results. The pose results were visualized in Schrödinger Maestro [54] and labeled in Inkscape. The pose results were analyzed for accuracy through the RMSD of the common core to the original cocrystal ligand core, calculated using Schrödinger’s Python API and visualized in matplotlib [72].

Schrödinger LigPrep was used to convert ligand SMILES using standard settings into Maestro structures for docking [75]. Glide’s cross-docking script, xglide.py, was used to perform ensemble docking for each clustering method. The cross-docking script generated receptor grid files for each centroid structure using a 32 Å box centered on the center of mass of the crystal structure’s ligand (BC7 [15]) to define the docking region. Each centroid was then docked to using Glide’s Standard Precision (SP) docking methodology, which includes its own ligand conformer generation steps, and scored with the subsequent Standard Precision GlideScore scoring function [50, 74]. For each ensemble docking approach, the best score of each ligand across the ensemble of conformations (**N**) was used to determine its rank,

1 $$\begin{aligned} s_l = \min \{s_{l,i}: \quad i \in \mathbf {N}\}, \end{aligned}$$

where $s_l$ and $s_{l,i}$ are the best overall score and best score for receptor conformation *i* for ligand *l* respectively.

To further investigate the ligand binding we also (1) applied a restraint on the tetrahydropyrido-pyrazole common core structure, restricted to lie within 3.5 Å of the cocrystal ligand’s common core, (2) changed the precision of the docking and scoring function from Glide SP to Glide Extra Precision (XP) [76], and (3) clustering and docking to centroids from a holo MD trajectory.

### Scoring schemes for ligand scores

Aside from changing the docking methodology from Glide SP to XP, we also compared the average and the weighted average (Eq. 2) of the Glide SP and XP scores.

2 $$\begin{aligned} s_l = \sum _{i \in \mathbf {N}} P_i \times s_{l,i}. \end{aligned}$$

where $P_i$ is the probability of observing conformation *i*, $s_{l,i}$ is the best docked score for that conformation, and $\mathbf {N}$ is the set of conformations in the ensemble. Note that the probabilities, $P_i,$ are normalized such that $\sum _{i \in \mathbf {N}}P_i = 1.$ The $P_i$ for a given conformation *i* is calculated as $f_i/f_T,$ where $f_i$ is the number of frames in the same cluster as *i*, and $f_T$ is the total number of frames in the trajectory. These scoring schemes have been used in other studies due to the reasoning that the average [37, 77] or weighted average [38, 39] score better accounts for the variability of the ensemble, and in the case of the weighted average, represents the likelihood of the ligand encountering each representative conformation in a natural environment.

### Kendall rank correlation coefficient for ligand rankings

Ligand rankings were created by sorting the ligands based on their Kendall rank correlation coefficient, also known as Kendall’s *τ* [78]. The Kendall’s *τ* coefficients were calculated by comparing the predicted rank ordering to the experimental rank ordering using the Kendall’s *τ* function in SciPy [79].

## Results and discussion

In lieu of running expensive free energy calculations which account for both ligand and receptor flexibility, the Relaxed Complex Scheme attempts to reduce computational cost while capturing the flexibility of a protein by docking to multiple protein conformations selected from a MD simulation. These representative conformations are often chosen by combining a method of dimensionality reduction followed by the application of a clustering algorithm. Although this approach is conceptually simple, the choice of clusters has many pitfalls. For example, even if a set of clusters spans the conformational diversity of the MD trajectory, the ensemble will not necessarily produce the most accurate ligand rank ordering [44]. Some receptors may have natural conformations which are not ideal for ligand binding, and these may result in false positives [29]. In addition, the active conformation for ligand binding could be transient, and would have a lower probability of being represented in the ensemble.

To observe the effect of clustering approaches on the resulting conformations, we test several different clustering methods. TICA captures slow protein movements (variance in time), while PCA focuses on large structural variance, and GROMOS captures structural variations as measured by RMSD. We plot the structural variation across clusters from the different algorithms in Fig. 2B, C.

We find that the clustering methods produce distinct centroid conformations, capturing structural variation in different areas of the protein. This is highlighted in Fig. 2C, where the residue RMSF of the 10 centroids from each clustering method are displayed by relative thickness and color. For example, the GROMOS centroids show substantial variation in the beta-sheet turn at the base of the protein (residues ARG 201 to ASN 205), especially when compared to PCA centroids (Fig. 2C). All centroids show some variation in the loop in the upper right of the protein (residues ALA 95 - GLN 101).

However, because structural fluctuation around the ligand binding site (facing the reader in Fig. 2A,C) is most likely to affect binding and is therefore of particular interest, we restricted the input feature set for clustering to the the atoms in the ligand binding site (Fig. 2A). Ligand binding site atoms were selected by taking all atoms within 2 Å of a CatS ligand from the D3R dataset that was docked using Glide SP. We refer to use of this restricted feature set in clustering as “Clustered by Binding Atoms” (CBA). The pairwise RMSD of all clustering methods, including CBA, is compared using a heatmap in Fig. 2B. Indeed, we find that restricting the input feature set to the ligand binding site atoms allows the clustering methods to capture increased variability at the site of interest. In Fig. 2C, for instance, TICA CBA (second row) shows more variation in the ligand binding site than the original TICA clustering using the backbone atoms (first row). Binding site variation is primarily due to the conformation of residue PHE 71, which is within the loop with high variation in TICA CBA (residues GLY 63 to PHE 71). PHE 71, along with PHE 212, frames the ligand binding site and changes the pocket landscape, which may affect ligand docking.

**Fig. 3:**
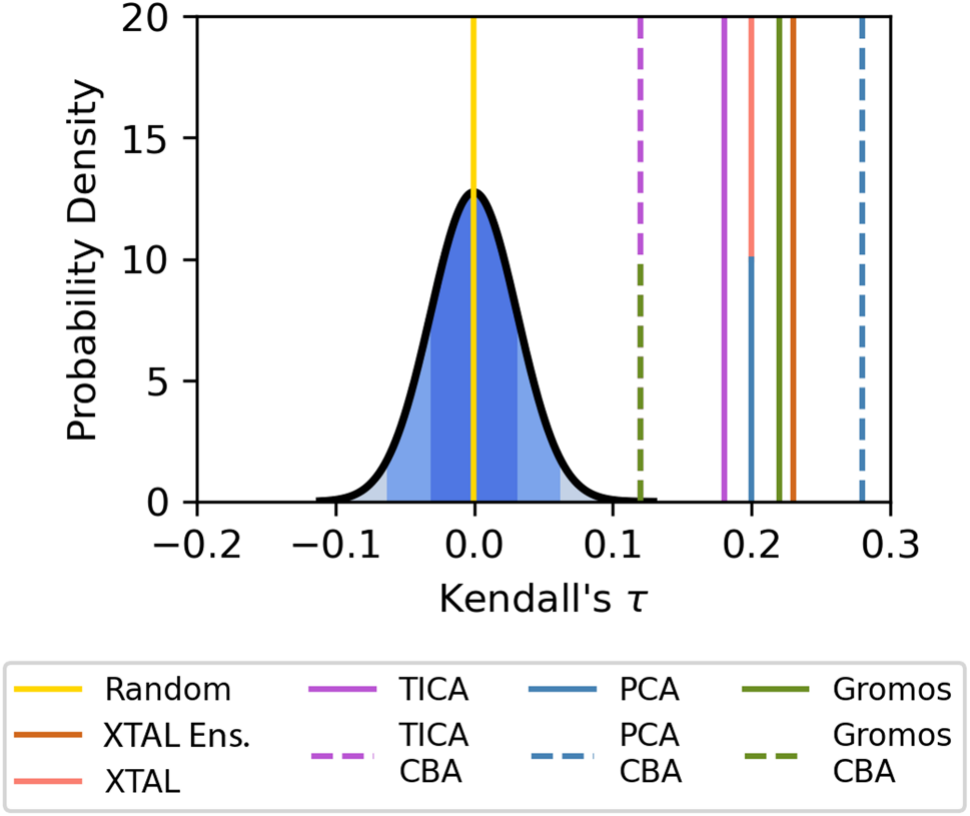
Kendall’s *τ* values for ligand rankings based on minimum scores from Glide docking to apo MD centroids, compared to a random rank ordering distribution. ‘XTAL Ens.’ indicates the crystal ensemble results. The probability distribution function is graphed from the Kendall’s *τ* values of 10,000 random ligand rank orderings. The distribution has *μ* = 0 and $\sigma = 0.031$

### Initial docking and scoring scheme results

We found that the rank order correlation of the predictions from Glide docking were better than random rank ordering (Fig. 3B).

**Table 1:** Kendall’s For All Ligand Rankings, The Kendall’s *τ*s for the initial Glide docking show slight fluctuations in different scoring schemes, but do not show any immense improvement

Docking function	Scoring method	Clustering methods						
		XTAL	TICA	PCA	GROMOS	TICA CBA	PCA CBA	GROMOS CBA
XTAL Ens. AB	Minimum	0.23						
	W. Avg.	–						
	Avg.	0.27						
Glide SP-AB	Minimum	0.20	0.18	0.18	0.22	0.12	0.28	0.12
	W. Avg.	–	0.21	0.20	0.18	0.17	0.24	0.21
	Avg.	–	0.20	0.21	0.25	0.20	0.24	0.23
Glide SP-AR	Minimum	0.13	0.14	0.13	0.13	0.11	0.11	0.09
	W. Avg.	–	0.08	0.07	0.10	0.09	0.05	0.07
	Avg.	–	0.12	0.07	0.09	0.07	0.06	0.09
Glide XP-AB	Minimum	0.20	0.11	0.11	0.12	0.11	0.24	0.14
	W. Avg.	–	0.10	0.08	0.08	0.11	0.17	0.15
	Avg.	–	0.11	0.08	0.07	0.12	0.19	0.17
Glide XP-AR	Minimum	0.13	0.14	0.10	0.12	0.11	0.09	0.13
	W. Avg.	–	0.10	0.07	0.04	0.07	0.03	0.11
	Avg.	–	0.11	0.07	0.06	0.04	0.04	0.08
Glide SP-HB	Minimum	0.09	0.17	0.14	0.18	0.23	0.23	0.18
	W. Avg.	–	0.20	− 0.01	0.17	0.24	0.14	0.20
	Avg.	–	0.22	− 0.01	0.21	0.23	0.18	0.21
Glide SP-HR	Minimum	0.12	0.18	0.13	0.11	0.16	0.17	0.15
	W. Avg.	–	0.13	0.09	0.08	0.15	0.11	0.13
	Avg.	–	0.15	0.11	0.12	0.14	0.13	0.14

Here we show the Kendall’s *τ* from rank orderings produced through various docking functions, clustering methods, and scoring schemes. Docking Functions are labeled accordingly: *XTAL Ens* Crystal Ensemble, *SP* Glide Standard Precision Docking, *XP* Glide Extra Precision Docking, *A* apo structure, *H* holo structure, *B* blind docking, *R* restrained docking. We experimented with these scoring schemes to test if a particular method of discerning scores for each ensemble would better represent the protein binding mechanisms and improve rank ordering. The various scoring schemes were the Minimum, Weighted Average (W. Avg.), and Average (Avg.)

Next, we investigated if the approach to compute a single score from an ensemble of scores can improve the accuracy of our predictions. There are several ways to obtain a single score from an ensemble of values. The first is to take the minimum score of the ensemble. This assumes that the other configurations do not contribute to the ligand binding energy. Relaxing this assumption, it is possible to consider the contributions of other receptor configurations by using an average or weighted average of the ensemble values. The choice of weights may be assigned by the probability of observing each conformation among other strategies. Limitations from the limited sampling of MD may lead to unintended biases in the ensemble weights.

In our results, we saw minor fluctuations in Kendall’s *τ*s across different scoring schemes (Table 1). While some conditions saw improvements to Kendall’s *τ* when using the weighted average versus the minimum score, no consistent rationale for these improvements were found. It is therefore unclear from this system and study whether or not incorporating receptor flexibility can improve predictions of rank ordered correlation. We hypothesize that the challenges of docking to CatS which has a large solvent-exposed binding pocket may outweigh the benefits of incorporating receptor flexibility which has been reported in other works [30, 34].

To further understand the shortcomings in our approach, we conducted multiple revisions to both the trajectory clustering and the docking methodology.

### Pose analysis and glide docking revisions

We found that the ligands in the CatS dataset had a common tetrahydropyrido-pyrazole core to other ligands with published cocrystal structures from a prior D3R Grand Challenge (GC3) (Fig. S3) [11, 53]. Other cocrystals contain ligands bound to this alternative site, although these ligands are dissimilar to the ones in our dataset (ligands 29 to 48 in Fig. S3, Table S2) [82].

**Fig. 4:**
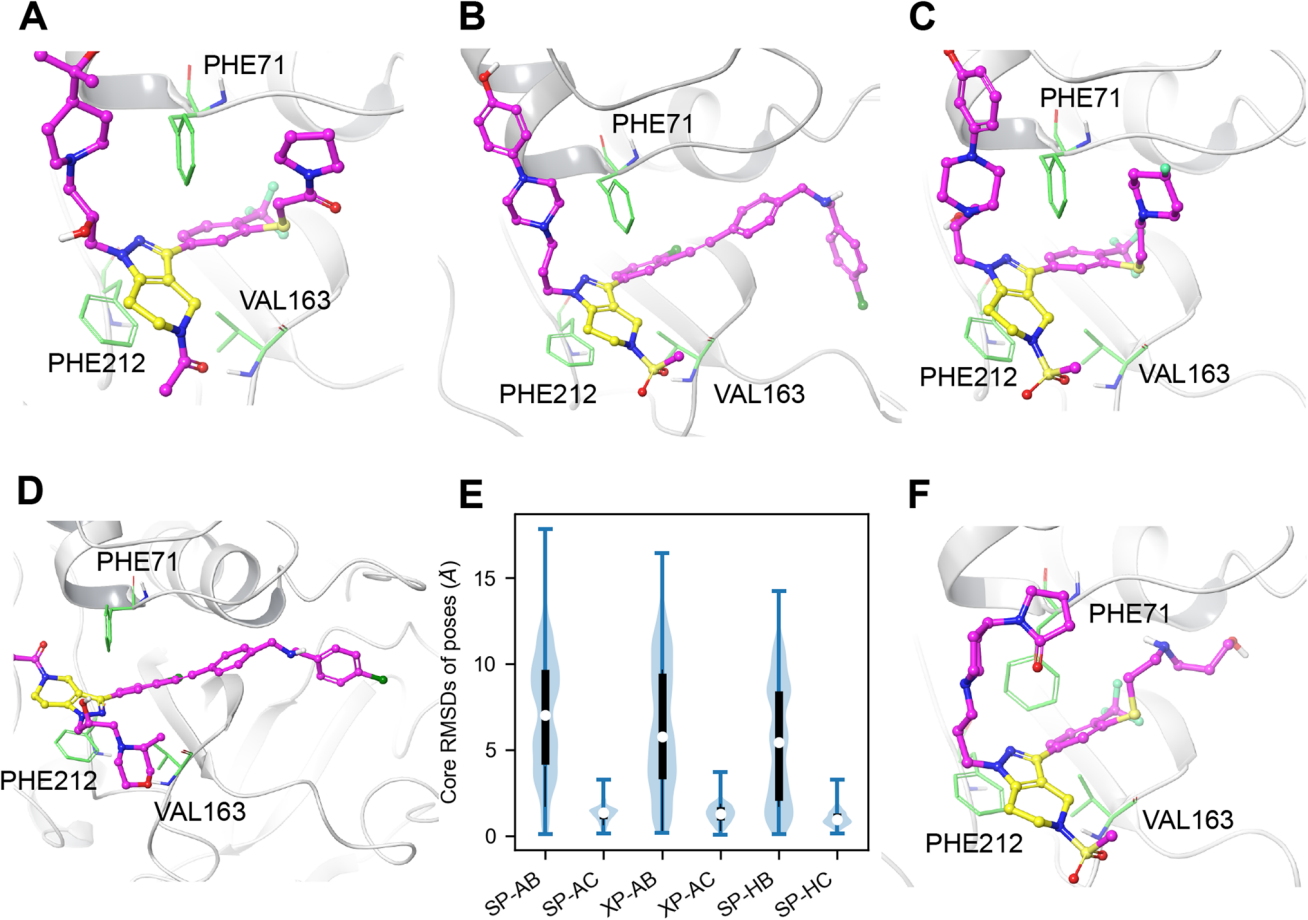
Docking pose analysis shows that a distance-restraint improves pose accuracy. **A** Cocrystal pose (PDB ID: 5QC4 [15]) Ligand carbons are pink; ligand common core carbons are yellow; key binding residues PHE71, VAL163, and PHE212 are green. **B** Ligand CatS 259 of the crystal ensemble docking: an ideal docking pose in an initial cocrystal structure. **C** Ligand CatS 118 of the SP apo blind crystal docking: ideal pose most similar to the cocrystal structure. **D** Ligand CatS 363 of the SP apo blind crystal docking: some docked ligands show a flipped core binding mode that is less common but can be found in some available cocrystals. [11]. **E** The RMSDs of the ligand core for each pose in each Glide docking method show that blind poses were concentrated farther from the cocrystal position compared to the ligand-core-restrained docking. Each violin is composed of all minimum poses for each clustering method which contributed to the final rank ordering and the crystal structure poses, totaling $n = 3213$ per violin. Method acronyms: *SP* Glide Standard Precision Docking, *XP* Glide Extra Precision Docking; *A* apo structure, *H* holo structure; *B* blind docking, *R* restrained docking. The median is represented in white, the interquartile range is shown in black, and the minimum and maximum values are shown as whiskers. **F** Ligand CatS 23 of the SP apo restrained PCA docking: When the ligand is restrained, it can be unnaturally docked in receptors that are dissimilar to the cocrystal, such as here where the PHE71 is in a different configuration

Glide docking produced some poses similar to the cocrystal pose (Fig. 4C) although it also produced unexpected poses. We also observed cocrystals with ligands binding in the less common “flipped core” configuration, shown in Fig. 4D, reported in GC3 [11].

To test the hypothesis whether improved pose similarity to cocrystal structures can improve docking accuracy, we applied a distance-restraint to the common core of the ligands using the core position of published cocrystals with similar ligands as a reference point (Fig. 4A). Other work has found that approaches which use information from cocrystals such as template docking or restraints can improve pose accuracy [9–11, 83, 84]. The restraint employed eliminated poses which deviate significantly from the cocrystal pose while permitting the flipped configuration. As shown in Fig. 4E, the RMSDs of the tetrahydropyrido-pyrazole core in SP apo docking were reduced (from a median of 7.39 Å to 1.40 Å) by adding the restraint, however, this did not improve the accuracy of the ranking (Table 1).

The ligand core restraint may not be appropriate for all centroids (e.g., Fig. 4F). The reference for the restraint is defined for all receptor configurations by RMSD alignment to the cocrystal structure. Receptor configurations which exhibit large structural differences from the cocrystal structure may have poor binding site alignment which introduces uncertainty into the approach. For some receptor configurations, restraints lead to atypical binding poses with high solvent accessibility.

To test whether using a more complex scoring function and search algorithm at the cost of computational efficiency can improve the predicted rank ordering, we compared between GlideScore SP and GlideScore Extra Precision (XP), with and without the restraint, using the same ensembles from apo MD. Compared to Glide SP, Glide XP (i) has more exhaustive docking by performing Glide SP docking then performing a separate anchor-and-grow sampling procedure, and (ii) the Glide XP scoring function penalizes ligand poses more harshly with desolvation penalties, identification of enhanced binding motifs, and higher receptor-ligand shape complementarity [76]. Glide XP has been found to outperform other methods and achieve better drug discovery results than Glide SP [85]. We found that using Glide XP for apo MD ensemble docking did not improve our rank-order predictions (Table 1), but the docked poses predicted by XP were more similar to the cocrystallized poses (Fig. 4E).

To test whether conformational selection may lead to improved results, we docked to centroids picked from a holo MD simulation. McGovern and Shoichet have shown that use of a holo structure can improve enrichment of lead compound identification [86]. We also expected that structures with a ligand would lead to lower ligand core RMSDs with more accurate active residue positioning. However, the Kendall’s *τ*s of the rank ordering stayed within the same range as the original apo docking, even when the ligand was restrained (Table 1). Upon further analysis of the structural fluctuations of the apo and holo MD centroids, we find that residue PHE71 is restricted by the ligand while other regions of the binding pocket exhibited similar structural variability (Fig. S4). As with the apo Glide SP docking results, when ligands were blindly docked using Glide SP to the holo structures the resulting docked poses were different than the cocrystal poses. The average RMSD of the docked ligand cores was 5.47 Å from the core of the cocrystal ligand (Fig. 4E). Overall, the blind docking to structures from the holo MD trajectory had a lower ligand core RMSD compared to the results from docking to the apo MD (Fig. S6). Ensemble docking with holo MD structures may improve performance because the ligand maintains a preferred binding site conformation throughout the simulation. When a core restraint was applied upon docking to configurations from the holo trajectory, the rank ordering did not improve (Table 1). Although others have suggested that improved poses could yield better scores [87], we found that improvements to the predicted poses from the application of ligand restraints and/or docking to holo receptor conformations did not improve our predictions. One explanation for the fact that holo MD ensemble constrained docking does not improve rank correlations compared with holo MD ensemble unconstrained docking may be that the structure already has the preferred ligand binding site conformation, and therefore improving the ligand pose will have a negligible effect. However, during comparative structure analysis we were not able to identify specific features to support this hypothesis.

### Crystal structure ensemble docking

Finally, to see if ligand rankings could be improved by docking to an ensemble of crystal structures instead of MD-derived structures, we docked the 459 CatS ligands to an ensemble of 55 CatS crystal structures from the PDB (Table S2, in addition to PDB ID 1GLO). To assess overall protein structural similarity, the pairwise RMSD within the crystal ensemble is plotted in Fig. S1, and the pairwise RMSD between the crystal ensemble and the apo and holo MD centroid structures is plotted in Fig. S2. The similarity of the cocrystal ensemble ligands to the 459 CatS ligands was compared by computing pairwise Tanimoto coefficients using RDKit topological fingerprints for each ligand [88]. The coefficients range from 0 (completely dissimilar) to 1 (identical), and are plotted in Fig. S3.

We found that the rank order correlation of the predictions from Glide docking to the crystal ensemble were better than random rank ordering and within the same range as the MD ensemble docking (Fig. 3B, Table 1). The correlation between docking scores and pIC50s was computed for the crystal ensemble (Pearson’s r = − 0.36) and was compared to the following conditions: (1) docking to the single CatS crystal structure PDB ID 5QC4 (r = − 0.25); and Glide SP docking to apo MD centroids that were clustered using (2) TICA (r = − 0.28), (3) PCA (r = − 0.39), and (4) GROMOS (r = − 0.31) (Fig. S7). In all conditions there is a weak negative correlation between docking score and pIC50. The correlation coefficients indicate that neither docking to the single crystal structure nor docking to the crystal structure ensemble indicates any obvious improvement over any MD-derived ensemble docking.

## Conclusion

In this work we describe our submission to subchallenge 2 of the Drug Design Data Resource (D3R) Grand Challenge 4 where we performed ensemble docking to rank order ligands by binding affinity. A comprehensive comparison of other participants’ methods, which performed substantially better than our naive approach, is available in the D3R Grand Challenge 4 manuscript [12]. Here, we explore and compare several factors including the choice of clustering algorithm for choosing representative receptor conformations and two docking workflows with and without restraints to improve pose accuracy. The different clustering algorithms produce different structural ensembles which can influence the docking results. Owing to the difficulty of docking to the CatS system, which has been recognized by others [89], we find that more sophisticated approaches can improve rank ordering compared to naive settings produced by GLIDE using a basic ensemble docking workflow [11]. We conclude that confounding factors and complications of the CatS system outweigh the benefits of ensemble docking. We explored if rank-order correlation could be improved with better pose accuracy by performing docking with restraints in addition to docking with receptor conformations extracted from a holo trajectory with ligand removed. We find that both approaches improve the pose similarity of docked ligands to related cocrystallized ligands, but do not improve the rank order correlation. Lastly, we demonstrate that docking to an ensemble of MD-generated structures does not yield improved rank-order correlations when compared with docking to an ensemble of crystal structures.

This project illustrates the benefits of partnering with high school and undergraduate students to participate in community challenges. Grand challenges are excellent resources for teaching research skills through a semi-guided, goal-oriented project, with expert curated datasets and deadlines. The students were exposed to important research skills, such as managing time, selecting and performing data analyses, and making publication-quality figures, at early stages of their scientific career. Owing to the computational nature of this challenge, the students also gained experience with data management, computational thinking, and script development. We suggest that student participation in community challenges can benefit both the community and the students and hope this work encourages others to explore this approach.

## Supplementary Material

The structural similarity of the dataset ligands to cocrystallized ligands, RMSF across receptor structures for the apo and holo trajectories, and comparison of ligand core RMSD across clustering methods are available in the supplemental information.

## Supplementary Information

Below is the link to the electronic supplementary material.

Supplementary file 1 (pdf 1184 KB)
