# Supplementary material for: Benchmarking ensemble docking methods in D3R Grand Challenge 4
Source: J Comput Aided Mol Des. 2022 Feb 24;36(2):87–99. doi: 10.1007/s10822-021-00433-2 (PMC8907095; doi:10.1007/s10822-021-00433-2)
Supplement: Supplementary file 1 — (pdf 1184 KB) [file 10822_2021_433_MOESM1_ESM.pdf]

Benchmarking ensemble docking methods in D3R Grand Challenge 4

Jessie Low Gan[1], Dhruv Kumar[2], Cynthia Chen[3,6], Bryn C. Taylor[4,7], Benjamin R. Jagger[4,8], Rommie E. Amaro[4,*], and Christopher T. Lee[5,*]

[1]San Diego Jewish Academy, San Diego, CA 92130
[2]Rancho Bernardo High School, San Diego, CA 92128
[3]Canyon Crest Academy, San Diego, CA 92130
[4]Department of Chemistry and Biochemistry, University of California San Diego, La Jolla, CA 92093
[5]Department of Mechanical and Aerospace Engineering, University of California San Diego, La Jolla, CA 92093
[6]Current Address: California Institute of Technology, Pasadena, CA 91125
[7]Current Address: Discovery Sciences, Janssen Research and Development, San Diego, CA 92121
[8]Current Address: Department of Bioengineering and Therapeutic Sciences, University of California San Francisco, San Francisco, CA 94158
[*]Correspondence: ramaro@ucsd.edu, ctlee@ucsd.edu

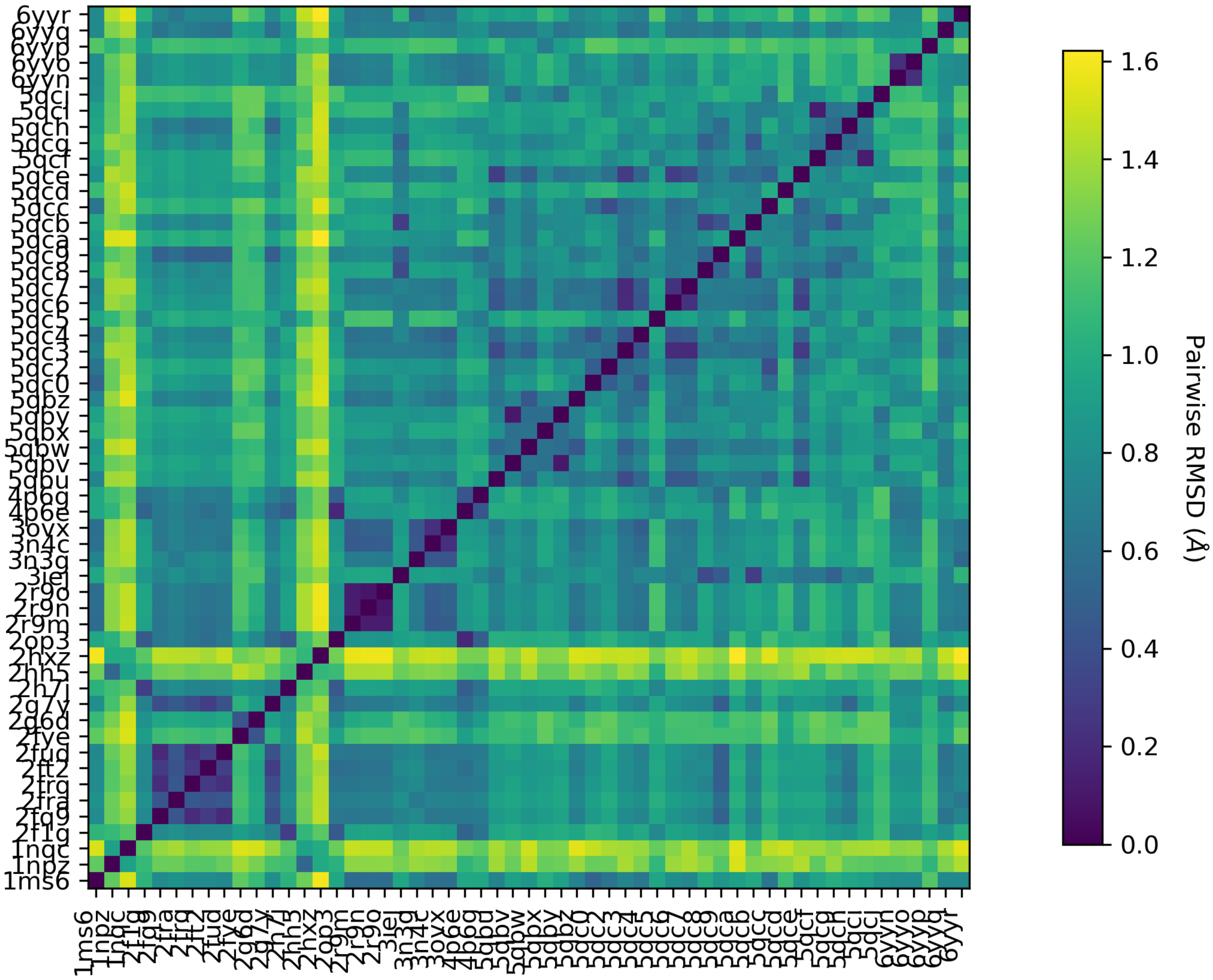

Figure S1: Pairwise RMSD values of the ensemble of 55 CatS crystal structures show the overall structural similarity. Structures were taken from RCSB PDB, and RMSD was calculated in MDTraj with the backbone atoms of the clustering-by-binding-atoms (CBA) selection, determined to be part of the binding site (Fig. 2) The heatmap was plotted in matplotlib (1).

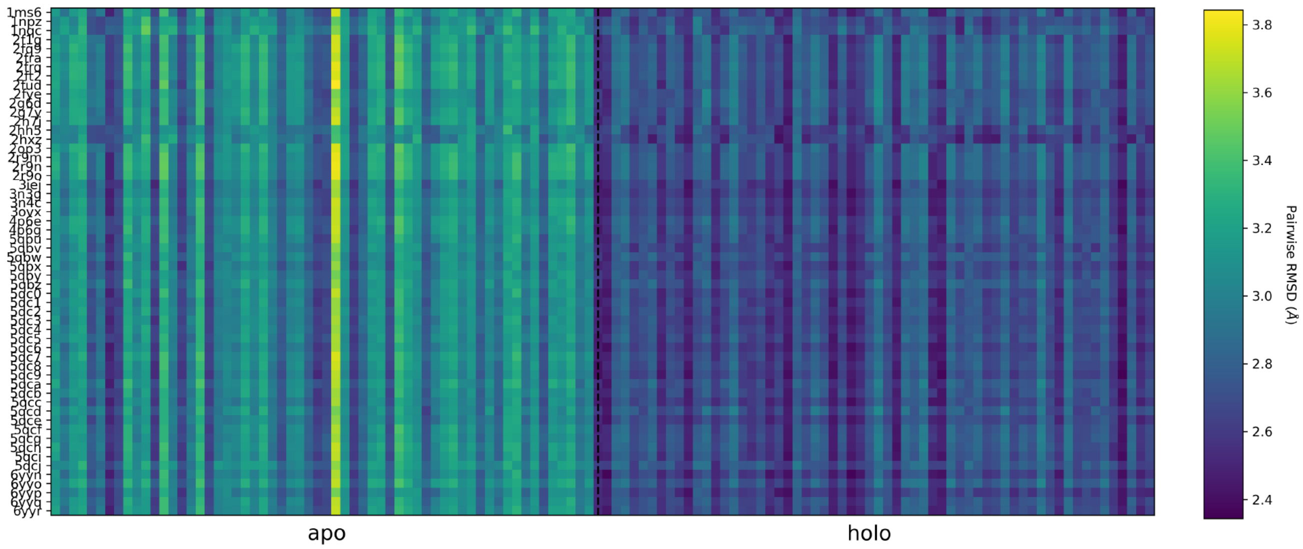

Figure S2: The pairwise RMSD values of the crystal ensemble compared to the centroids extracted from the MD trajectories show its similarity to an MD trajectory that was simulated as a cocrystal (holo) vs. a trajectory without a ligand (apo). Structures were taken from RCSB PDB, and RMSD was calculated in MDTraj with the backbone atoms of the clustering-by-binding-atoms (CBA) selection, determined to be part of the binding site (Fig. 2) The heatmap was plotted in matplotlib (1).

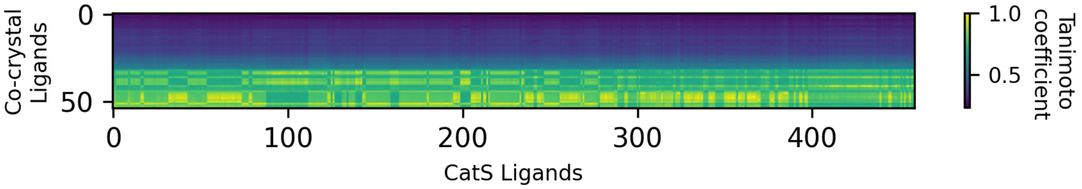

Figure S3: The pairwise Tanimoto coefficients of the 54 crystal ensemble ligands to the 459 CatS ligands highlights ligand similarities and differences, with Tanimoto scores ranging from 0 (completely dissimilar structures) to 1 (identical). 54 cocrystal ligands were downloaded from RCSB PDB, and Tanimoto coefficients were calculated using the RDKit Fingerprint implemented in RDKit (2). The corresponding PDB IDs are in Table S1, and the ligands are ordered by the L^2 norm of the Tanimoto coefficients, and plotted in matplotlib (1).

Table S1: The trajectory frame numbers of the centroids for each clustering methods. 5 replicate trajectories were run for apo and holo each, and all frames were considered in clustering, thereby cluster centroids were taken from any of the 5 replicates. Frames were not minimized upon extraction. Upon inspection, loops with the most structural variability include residues ARG201 to ASN205 and ALA95 to GLN101. Notably, binding site residue PHE71 shows fluctuations and affects the binding pocket landscape.

MD Trajectory	Centroid Number	apo Replicate Number	apo Trajectory Index	holo Replicate Number	holo Trajectory Index
TICA	0	0	8956	1	78892
	1	0	16806	3	77900
	2	0	77220	2	82008
	3	0	66191	1	64728
	4	3	63604	1	36285
	5	0	11040	3	240
	6	0	70083	1	35172
	7	4	28975	3	72160
	8	1	15084	0	49900
	9	0	4325	5	49135
TICA CBA	0	0	56621	1	9143
	1	0	74395	0	51588
	2	0	52127	0	49378
	3	0	19707	2	5516
	4	0	18031	1	59076
	5	1	151	2	20735
	6	0	6343	0	44332
	7	0	25990	1	77008
	8	0	64821	4	71640
	9	0	41814	1	7735
PCA	0	0	88481	0	82249
	1	0	14867	0	60176
	2	0	40217	0	77534
	3	0	7752	0	89983
	4	0	35642	1	5982
	5	0	43653	1	36342
	6	0	49902	1	12122
	7	0	29965	1	78834
	8	3	37784	3	67304
	9	2	32804	5	46350
PCA CBA	0	4	60400	0	74880
	1	0	11730	0	55396
	2	0	79440	0	43977
	3	0	25687	0	51981
	4	0	40390	0	6973
	5	0	27488	1	44740
	6	0	7484	3	4536
	7	1	3633	0	40528
	8	1	64528	3	22580
	9	1	54032	4	65540
GROMOS	0	1	68300	3	67400
	1	1	19800	2	29000
	2	3	45800	3	86100
	3	2	32400	4	62300

	4	2	17700	2	32500
	5	1	24900	3	54600
	6	0	11700	4	22400
	7	3	53600	2	86000
	8	4	15900	3	53600
	9	1	89500	1	70300
GROMOS CBA	0	3	23300	3	59700
	1	2	7300	2	11300
	2	1	5800	0	33500
	3	3	15800	2	18500
	4	3	45500	4	88500
	5	4	70900	4	68600
	6	3	13300	2	7700
	7	2	58900	2	82100
	8	4	87500	1	73200
	9	0	47800	0	71600

Table S2: Corresponding PDB IDs from the RCSB PDB database to co-crystal numbers from Fig. S1. Ordered by the L^2 norm of pairwise Tanimoto coefficients between cocrystal ligands and 459 CatS ligands. Crystal structure PDBID 1GLO did not have a cocrystal ligand and is therefore not included in this table.

cocrystal Number	PDBID	Ligand ID	L^2 Norm
0	5qc8	BFV	18.05
1	5qcf	BJD	17.95
2	5qca	BGJ	17.9
3	5qc6	BCJ	17.79
4	5qci	BJV	17.64
5	5qcg	BJJ	17.46
6	5qcd	BHJ	17.33
7	5qc1	B9S	17.31
8	5qc5	BAJ	17.13
9	5qcj	BJY	16.88
10	5qch	BJS	16.73
11	5qc3	B9Y	16.67
12	3iej	599	16.64
13	5qc4	BC7	16.53
14	5qbx	B8V	16.47
15	5qc0	BQJ	16.24
16	5qc2	BQP	16.22
17	5qc7	BQS	16.22
18	5qcb	BHV	15.83
19	5qc9	BG7	15.78
20	5qcc	BGY	15.57
21	5qbu	B8J	15.28
22	5qbv	N2D	12.21
23	5qbw	B8S	11.74
24	5qbz	B8Y	11.42
25	5qby	N2A	10.92
26	4p6g	2FZ	10.39
27	3n4c	EF3	10.23
28	2fye	BCQ	9.83
29	2h7j	H7J	9.21
30	6yyr	Q1N	8.7
31	2r9o	C28	8.35
32	2g7y	MO9	8.1
33	2fq9	CRJ	8.09
34	2fra	CRV	8.06
35	2hh5	GNQ	7.86
36	2f1g	GNF	7.76
37	4p6e	2FC	7.75
38	5qbz	935	7.72
39	2r9n	Y14	7.69
40	2frq	C71	7.68
41	6yyp	Q1H	7.45
42	2g6d	MQQ	7.36
43	1npz	C1P	7.35
44	6yyn	Q1E	7.29
45	2fud	CRL	6.78
46	1nqc	C4P	6.66
47	2r9o	Y15	6.57
48	6yyo	Q1K	6.56

49	6yyq	Q1Q	6.29
50	2r9m	Y11	5.97
51	1ms6	BLN	5.91
52	5qc1	O64	5.85
53	2op3	TF5	5.1

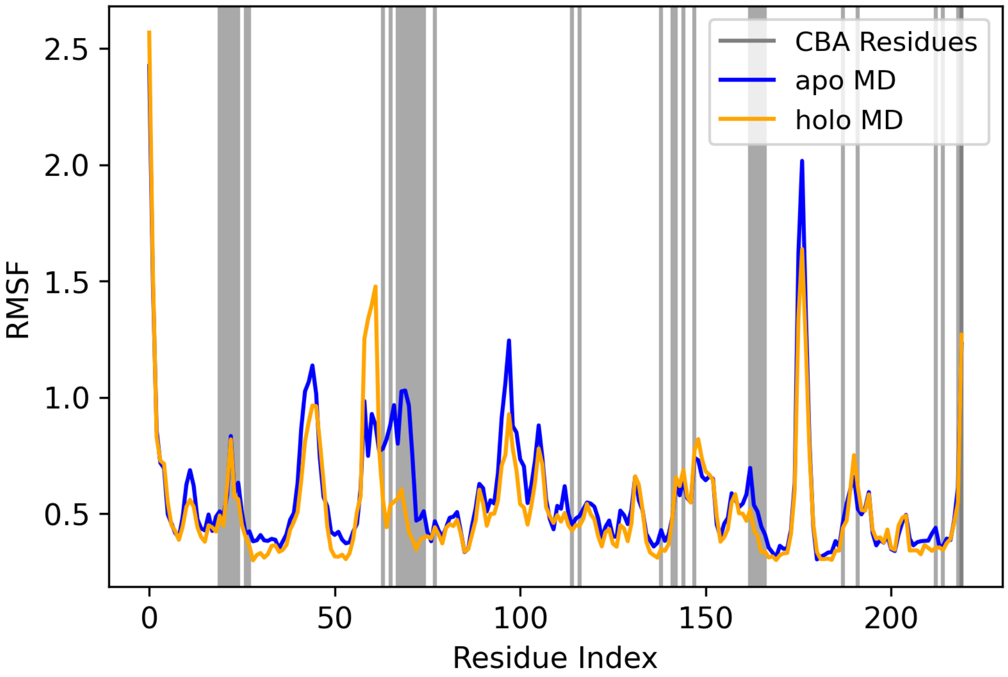

Figure S4: The RMSF of the apo MD and holo MD show slight structural variation in certain residues. RMSF was calculated in CPPTRAJ and plotted using matplotlib (1, 3). The residues that contain any clustering-by-binding-atoms (CBA), determined to be part of the binding site (Fig. 2), are indicated by vertical grey lines, and their indices were extracted with MDTraj (4).

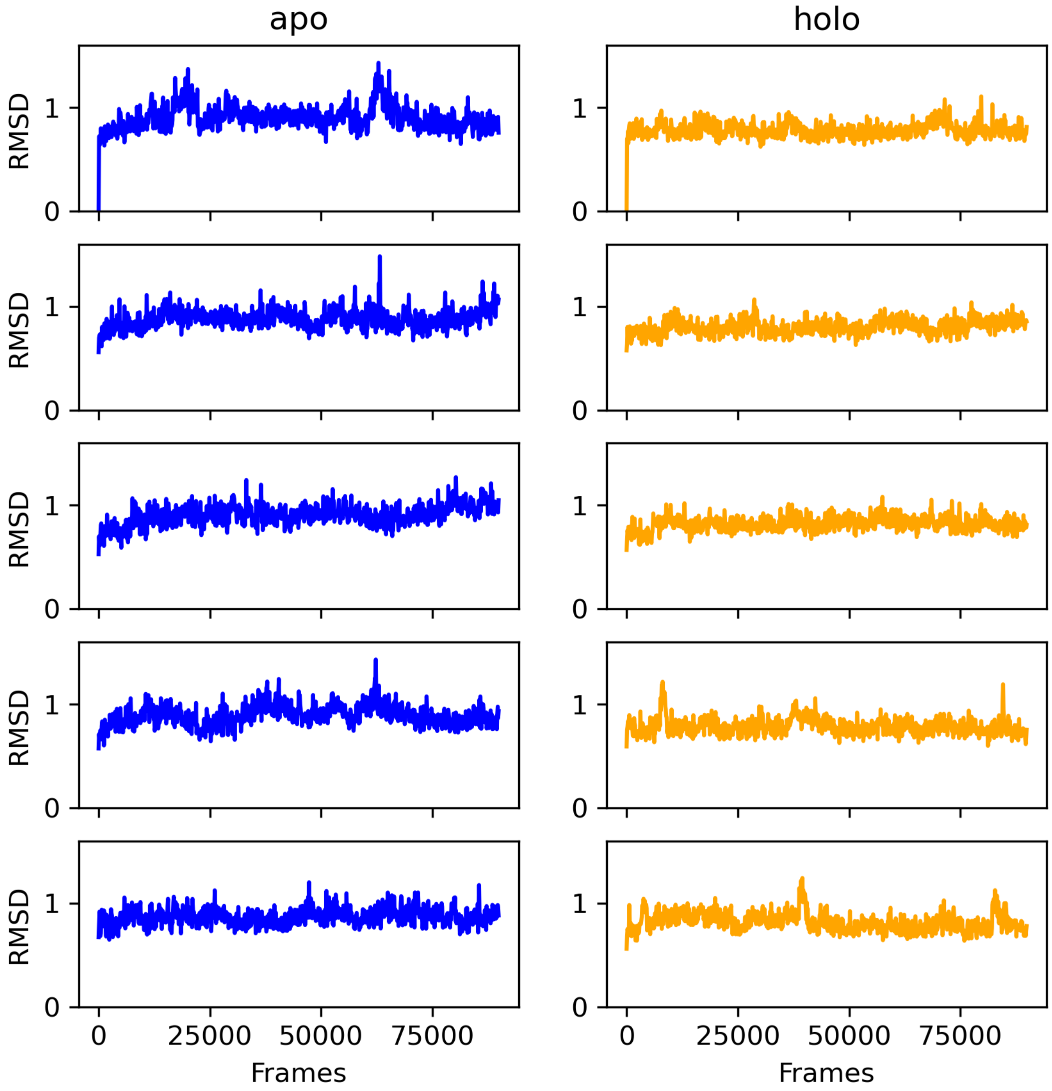

Figure S5: Apo and holo RMSD show slight structural variation over the trajectories. RMSD was calculated in CPPTRAJ over all the protein backbone atoms and every 100 frames, and was plotted with matplotlib (1, 3).

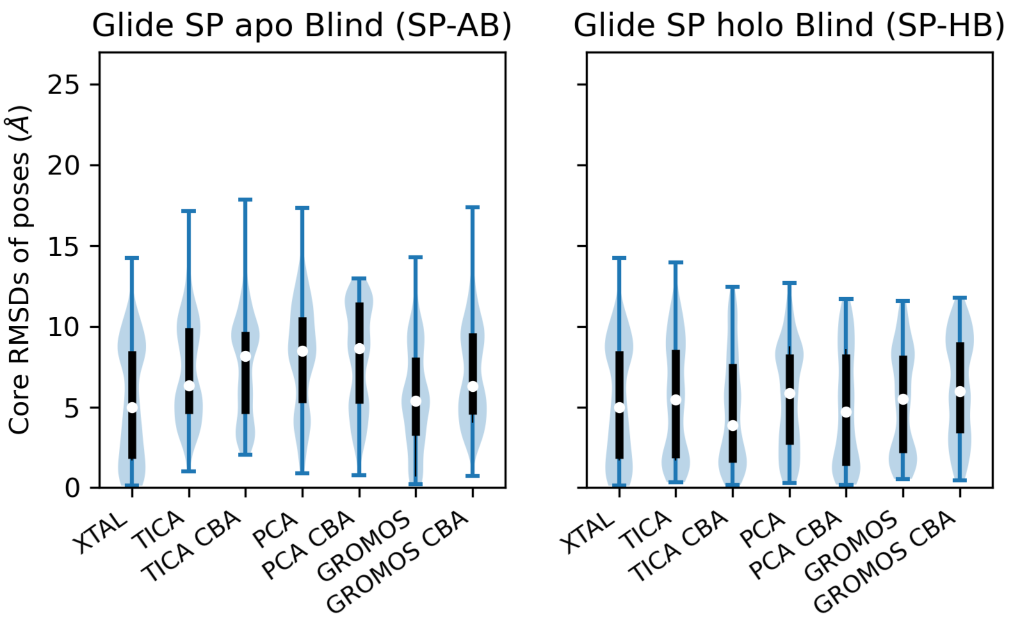

Figure S6: Ligand poses from holo MD docking are more similar to cocrystal ligand poses. The ligand core RMSDs for the Glide Standard Precision (SP) docking results of the apo blind (SP-AB) and holo blind (SP-HB) are expanded from Fig. 4, into the various clustering methods, where the median seems to be slightly lower when given the holo MD centroids. The ligand core RMSDs were calculated with Schrodinger's python API and plotted in matplotlib(1).

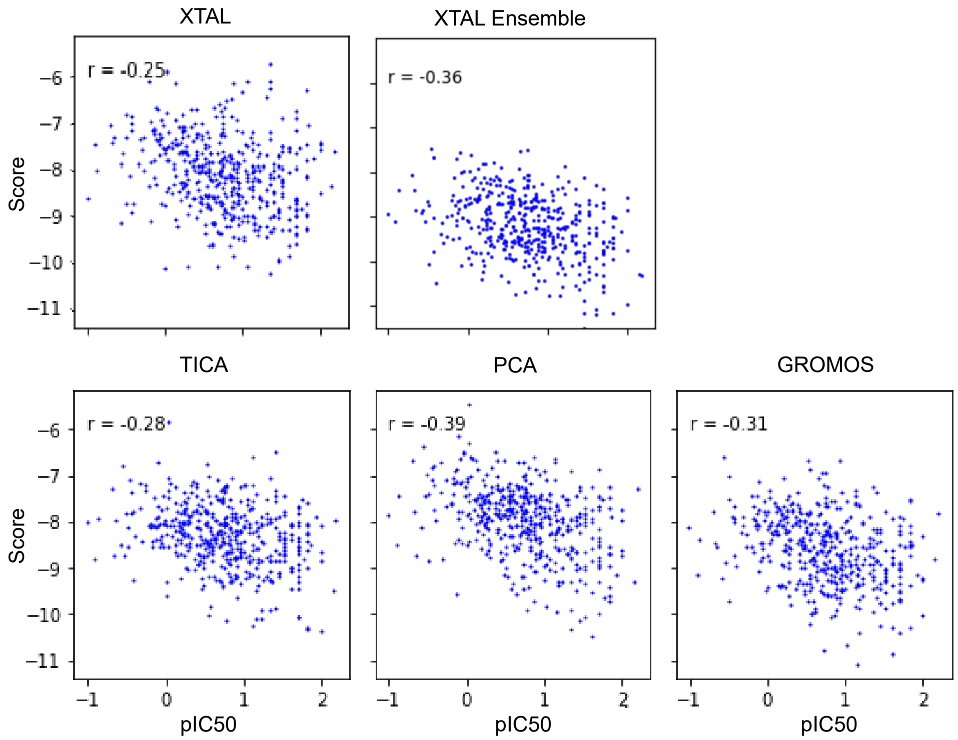

Figure S7: The 459 CatS ligands were docked to the crystal structure (XTAL), crystal ensemble (XTAL Ensemble), and MD-generated centroids derived from the 3 clustering methods (TICA, PCA, GROMOS; all Glide SP apo docking). We find that there is a weak negative correlation between docking scores and recorded pIC50s (Pearson's *r*) in all conditions, and that there is no improvement in correlation when docking to the crystal structure (XTAL) or crystal ensemble (XTAL Ensemble). Pearson's *r* was calculated with scipy(5) and the figure was plotted in matplotlib(1).

REFERENCES

1. John D. Hunter. “Matplotlib: A 2D Graphics Environment”. In: *Computing in Science & Engineering* 9.3 (2007), pp. 90–95. ISSN: 1521-9615. DOI: 10.1109/MCSE.2007.55.
2. “RDKit: Open-Source Cheminformatics”. In: ().
3. Daniel R. Roe and Thomas E. Cheatham. “PTRAJ and CPPTRAJ: Software for Processing and Analysis of Molecular Dynamics Trajectory Data”. English. In: *Journal of Chemical Theory and Computation* 9.7 (2013), pp. 3084–3095. ISSN: 1549-9618, 1549-9626. DOI: 10.1021/ct400341p.
4. Robert T. McGibbon et al. “MDTraj: A Modern Open Library for the Analysis of Molecular Dynamics Trajectories”. English. In: *Biophysical Journal* 109.8 (2015), pp. 1528–1532. ISSN: 00063495. DOI: 10.1016/j.bpj.2015.08.015.
5. SciPy 1.0 Contributors et al. “SciPy 1.0: Fundamental Algorithms for Scientific Computing in Python”. English. In: *Nature Methods* 17.3 (2020), pp. 261–272. ISSN: 1548-7091, 1548-7105. DOI: 10.1038/s41592-019-0686-2.
